# Femur-First Cut Kinematically Aligned Unicompartmental Knee Arthroplasty: A Seamless Surgical Technique from UKA to TKA

**DOI:** 10.1016/j.artd.2026.102147

**Published:** 2026-09-24

**Authors:** Yoshinori Soda, Mitsuhiro Nakamura, Toshiya Kano

**Affiliations:** a Hiroshima Midorii Joint Replacement Center, Saka Midorii Hospital, Hiroshima City, Japan; b Department of Orthopaedic Surgery, Hiroshima City Hospital, Hiroshima City, Japan

**Keywords:** Kinematic alignment, Unicompartmental knee arthroplasty, Fixed-bearing UKA, Femur-first technique, Surgical technique

## Abstract

Kinematic alignment (KA) is based on a resurfacing philosophy that restores the patient's native joint line orientation and preserves physiological soft-tissue balance. In calipered KA total knee arthroplasty, reconstruction of the distal femoral articular surface is the first and most critical step because it establishes the cylindrical axis that governs knee kinematics. Although unicompartmental knee arthroplasty (UKA) shares the same resurfacing philosophy, most currently available techniques begin with tibial resection, making femoral component positioning dependent on tibial alignment, gap balancing, and implant thickness. This may reduce the reproducibility of femoral component orientation and differs conceptually from established calipered KA principles. This Technical Note describes a femur-first calipered kinematically aligned technique for fixed-bearing medial UKA. The procedure reconstructs the distal and posterior femoral articular surfaces before tibial preparation using caliper verification of bone resections. Tibial resection is subsequently performed independently to reproduce the native posterior tibial slope while preserving physiological ligament tension throughout the range of motion. The described workflow follows the established principles of calipered KA while remaining applicable to fixed-bearing UKA. By establishing femoral anatomy before tibial preparation, this technique provides a reproducible and anatomically consistent resurfacing strategy and may facilitate intraoperative conversion to total knee arthroplasty when necessary.

## Introduction

Unicompartmental knee arthroplasty (UKA) is an established treatment for isolated medial compartment osteoarthritis and offers several advantages over total knee arthroplasty (TKA), including preservation of bone stock, maintenance of native ligament function, and faster postoperative recovery. Nevertheless, its worldwide utilization remains relatively low because of concerns regarding technical difficulty, reproducibility, and revision rates compared with TKA [1,2].

Kinematic alignment (KA) was developed for TKA as an individualized resurfacing technique that restores the patient's native joint line orientation and preserves physiological soft-tissue balance rather than correcting constitutional alignment [[3], [4], [5], [6]]. In calipered KA-TKA, the distal femoral articular surface is reconstructed first by compensating for cartilage wear and matching bone-resection thickness to implant thickness. Restoration of the femoral cylindrical axis provides the anatomical reference for subsequent bone preparation and soft-tissue preservation.

Although UKA is likewise a resurfacing procedure, most conventional surgical techniques begin with tibial resection. Consequently, femoral component positioning is determined secondarily according to the tibial cut, gap balancing, and insert thickness. While these techniques have demonstrated excellent clinical outcomes, the workflow differs from the established principles of calipered KA and may contribute to variability in femoral component orientation among patients [7,12].

Recent investigations have suggested that kinematically aligned UKA may improve restoration of native knee kinematics and provide favorable clinical outcomes [[8], [9], [10]]. Furthermore, the medial wall of the medial femoral condyle has been identified as a reliable anatomical landmark for femoral component alignment in KA-UKA [11]. These observations support reconstruction of femoral anatomy before tibial preparation.

The purpose of this Technical Note is to describe a femur-first calipered kinematically aligned technique for fixed-bearing medial UKA. The technique reconstructs the distal and posterior femoral articular surfaces before tibial preparation, verifies bone resections using caliper measurements, and reproduces the native posterior tibial slope while preserving physiological soft-tissue balance. Although demonstrated using one implant system, the surgical principles are intended to be applicable to fixed-bearing UKA in general.

## Rationale for the femur-first cut KA-UKA technique

UKA is, by definition, an articular surface resurfacing procedure rather than a corrective osteotomy. Accordingly, its alignment philosophy should be consistent with that of KA-TKA.

The cylindrical axis of the distal femur is the primary determinant of tibiofemoral kinematics throughout most of the range of motion [6]. Reliable anatomical landmarks, including the medial wall of the medial femoral condyle, have been identified for femoral component positioning in KA-UKA [11]. From this perspective, a tibial-first approach—which presumes an un-reconstructed femur—is inconsistent with KA principles.

When the tibia is resected first, femoral component alignment is indirectly dictated by tibial resection angle, gap tension, and polyethylene thickness. This often leads to substantial inter-patient variability in femoral coronal alignment, even among knees with similar femoral morphology [12]. Such variability is difficult to justify in a resurfacing procedure intended to restore native knee kinematics.

## Surgical technique

We present the surgical technique of femur-first cut calipered kinematically aligned medial UKA with the MOTO partial knee implant (Medacta Corporate, Castel San Pietro, Switzerland).

### Exposure and initial assessment

A tourniquet is applied to the proximal thigh. A skin incision is made using a limited medial parapatellar approach extending from approximately one fingerbreadth proximal to the superior pole of the patella to the tibial tubercle. The patella is gently shifted laterally to allow inspection of the medial compartment.

The anterior cruciate ligament (ACL), lateral compartment cartilage, and patellofemoral joint are assessed intraoperatively. If ACL deficiency is identified, the decision to proceed with UKA or convert to TKA is based on the overall intraoperative findings, including knee stability, condition of the remaining compartments, and surgeon judgment.

Medial and lateral osteophytes around the femur and tibia are removed. In cases with partial cartilage wear of the distal medial femoral condyle, the remaining cartilage is completely removed using a curette or bone saw to expose the native subchondral bone surface.

### Distal femoral resection

As in calipered KA-TKA, the distal femoral cortex is palpated, and an intramedullary rod is carefully inserted. Placement of the distal reference guide before rod insertion facilitates accurate positioning.

A distal femoral reference guide compensating for the thickness of the removed cartilage is applied to the cartilage-denuded medial femoral condyle. Distal femoral resection is then performed (Fig. 1).

**Figure 1:**
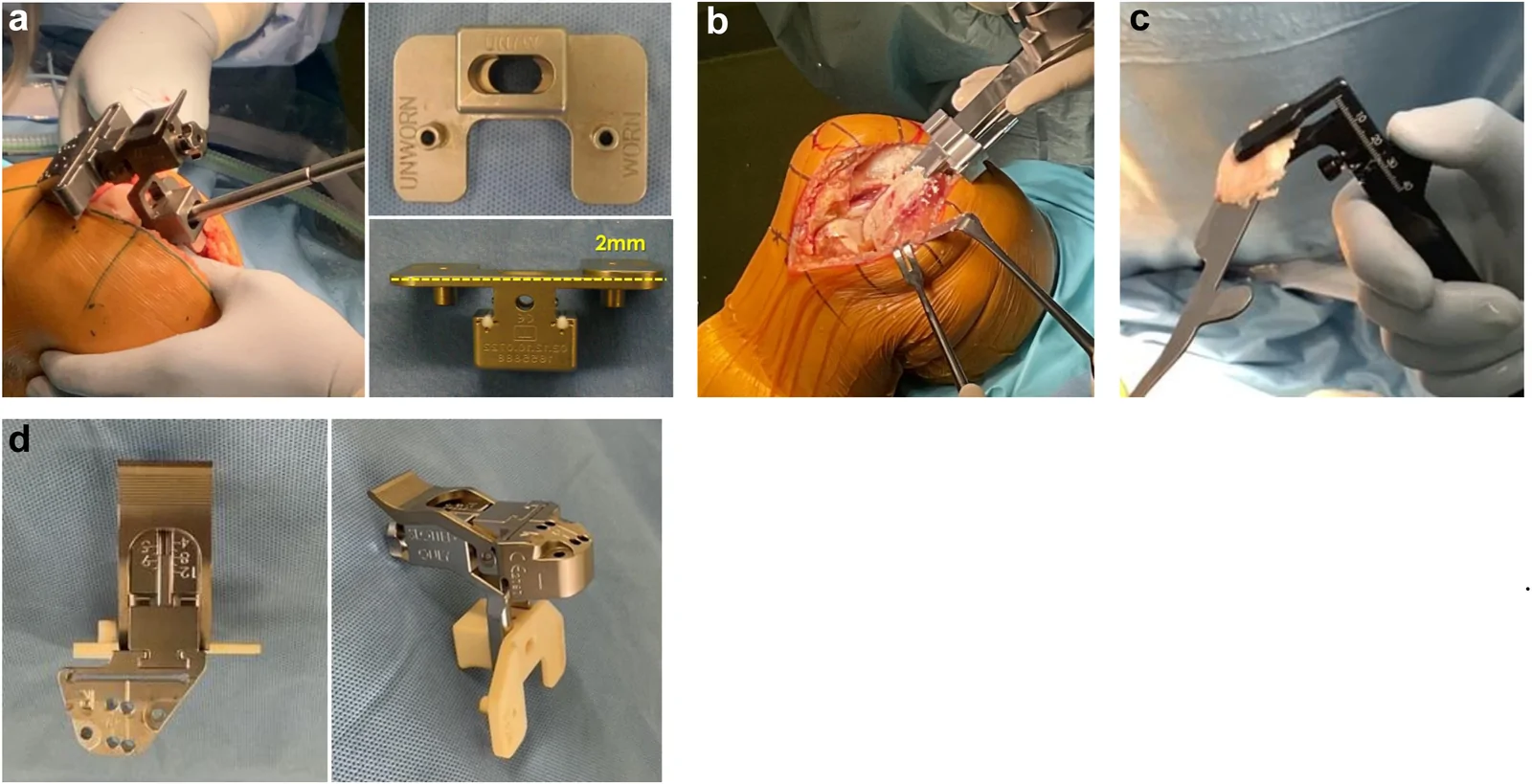
(a) A distal reference guide (standard instrument for TKA) compensating for 2 mm of cartilage thickness with metal augmentation is applied to the cartilage-denuded medial condyle. (b) Distal femoral resection is performed. (c) The resected bone thickness is measured with calipers. (d) Downsized instruments (under development).

The resected bone thickness is measured using a caliper and compared with the planned femoral component thickness. In accordance with calipered KA principles, the combined thickness of the resected bone, the estimated cartilage loss (approximately 2 mm), and the saw blade kerf should correspond to the femoral component thickness. The objective is to restore the native distal femoral articular surface by matching the amount of bone removed with the implant thickness.

If the resection is insufficient, additional bone resection is performed. If the measured resection is greater than that planned, the final assessment is performed using trial components to evaluate component position, stability, and soft-tissue tension. When appropriate, the discrepancy can be accommodated by selecting an alternative polyethylene insert thickness within the available implant options.

The final decision is therefore based not only on caliper measurement but also on intraoperative trial assessment to reproduce appropriate knee kinematics. (Currently, standard TKA instruments are used for distal femoral preparation; downsized instruments specifically designed for this procedure are under development).

### Femoral sizing and rotation

Because conventional UKA femoral sizers are generally designed for tibia-first procedures, femoral sizing is performed after reconstruction of the distal femoral surface and before tibial preparation.

A femoral sizing instrument is placed on the distal femoral cut surface. The anterior-posterior position and size are determined by confirming appropriate coverage of the reconstructed femoral surface while avoiding cortical overhang or insufficient implant coverage.

An angel wing is used to confirm that the sizing paddle contacts the most prominent point of the posterior femoral condyle (Fig. 2a).

**Figure 2:**
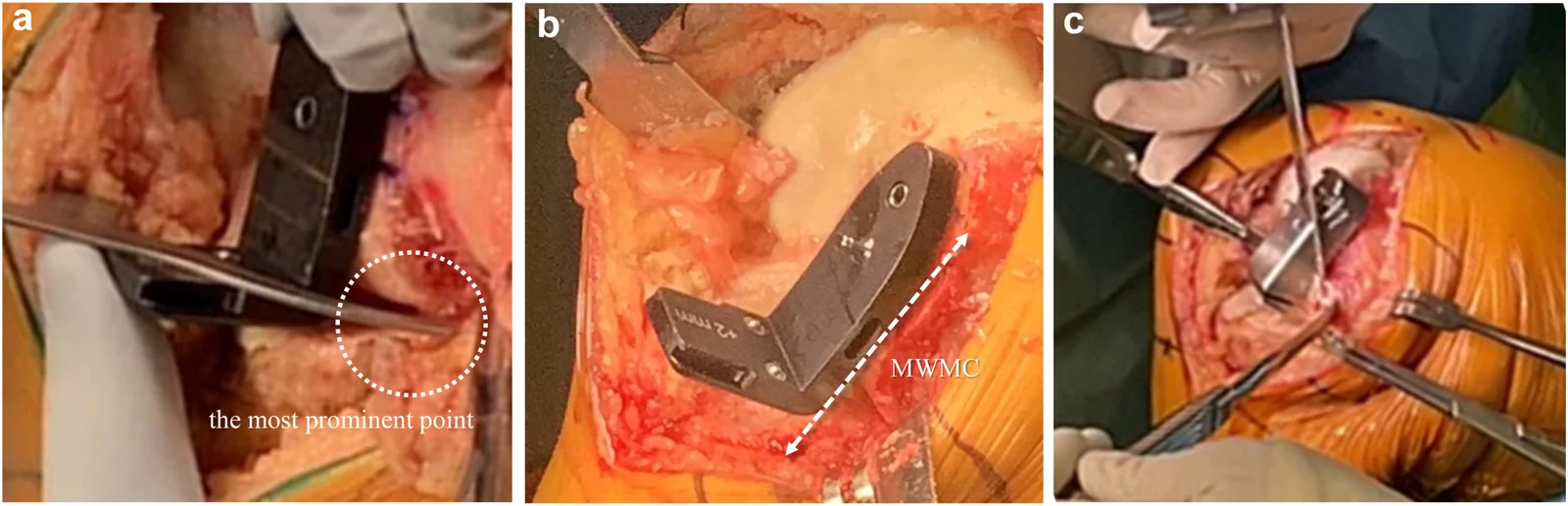
(a) A contralateral femoral sizer is inverted and placed directly on the distal femoral cut surface. Using an angel wing, the paddle surface of the femoral sizer is confirmed to contact the most prominent point of the posterior femoral condyle, and the size is determined. (b) Femoral sizer is set parallel to the medial wall of the medial femoral condyle (MWMC), based on the extension of the line connecting the midpoint of the posterior condylar articular surfaces. (c) The cutting block is then fixed, and femoral resections are completed.

Femoral rotation is determined according to the medial wall of the medial femoral condyle. The rotational reference is based on the extension of the line connecting the midpoint of the posterior condylar articular surfaces (flexion facet center) and the anatomical landmark described by Preston et al. (Fig. 2b).

Because the posterior femoral condylar reference is established before tibial preparation, femoral rotation is not influenced by tibial resection height or polyethylene thickness. The femoral cutting block is then fixed, and the remaining femoral resections are completed (Fig. 2c).

After the posterior femoral resections are completed, the resected bone thickness is measured using a caliper. Similar to the distal femoral resection, the combined thickness of the resected bone, residual cartilage loss (approximately 2 mm when full-thickness cartilage loss is present), and the saw blade kerf should correspond to the posterior femoral component thickness. When posterior cartilage is partially preserved, the measured bone thickness is interpreted after accounting for the remaining cartilage thickness. If caliper measurements differ from the expected values, component position is reassessed and confirmed using trial components and overall knee kinematics rather than caliper measurements alone.

### Tibial exposure and posterior tibial slope determination

Following completion of femoral preparation, adequate visualization of the medial tibial plateau is obtained.

The tibia is gently externally rotated and anteriorly translated. Because distal and posterior femoral resections have already been completed, anterior translation of the tibia can be achieved without additional ligament release.

A blunt Hohmann retractor is inserted through the intercondylar notch behind the proximal tibia, allowing controlled anterior translation while preserving the ACL and deep medial collateral ligament. The medial tibial plateau is exposed to the level required for implant preparation.

The medial meniscus is removed en bloc (Fig. 3a).

**Figure 3:**
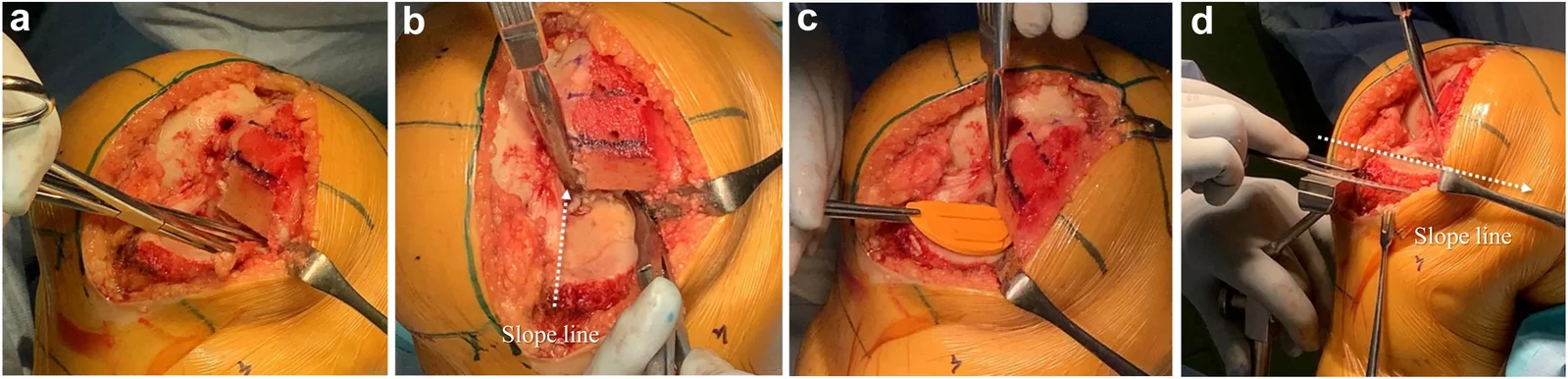
(a) The medial meniscus is excised en bloc. (b) An anteroposterior reference line (“slope line”) is drawn at the base of the intercondylar eminence. (c) This line can be determined based on tibial surface morphology and implant coverage. (d) Slope line is observed from the medial aspect of the knee, and the posterior slope of the tibial jig is set to reproduce the native posterior tibial slope, parallel to “slope line.”

An anteroposterior reference line (“slope line”) is marked at the base of the intercondylar eminence (Fig. 3b). This line is determined according to the native tibial surface morphology and the planned implant coverage rather than relying on a single fixed bony landmark (Fig. 3c).

After determining the appropriate tibial component size using trial components, the slope line is visualized from the medial aspect of the knee. The posterior tibial slope of the tibial cutting guide is then adjusted to reproduce the native posterior tibial slope, maintaining parallelism with the slope line (Fig. 3d).

### Tibial resection

With the knee in extension, manual in-line traction [[13], [14]] is applied to the lower limb (Fig. 4a). This traction method is used throughout tibial preparation as the reference condition for evaluating the relationship between the reconstructed femoral surface and tibial resection.

**Figure 4:**
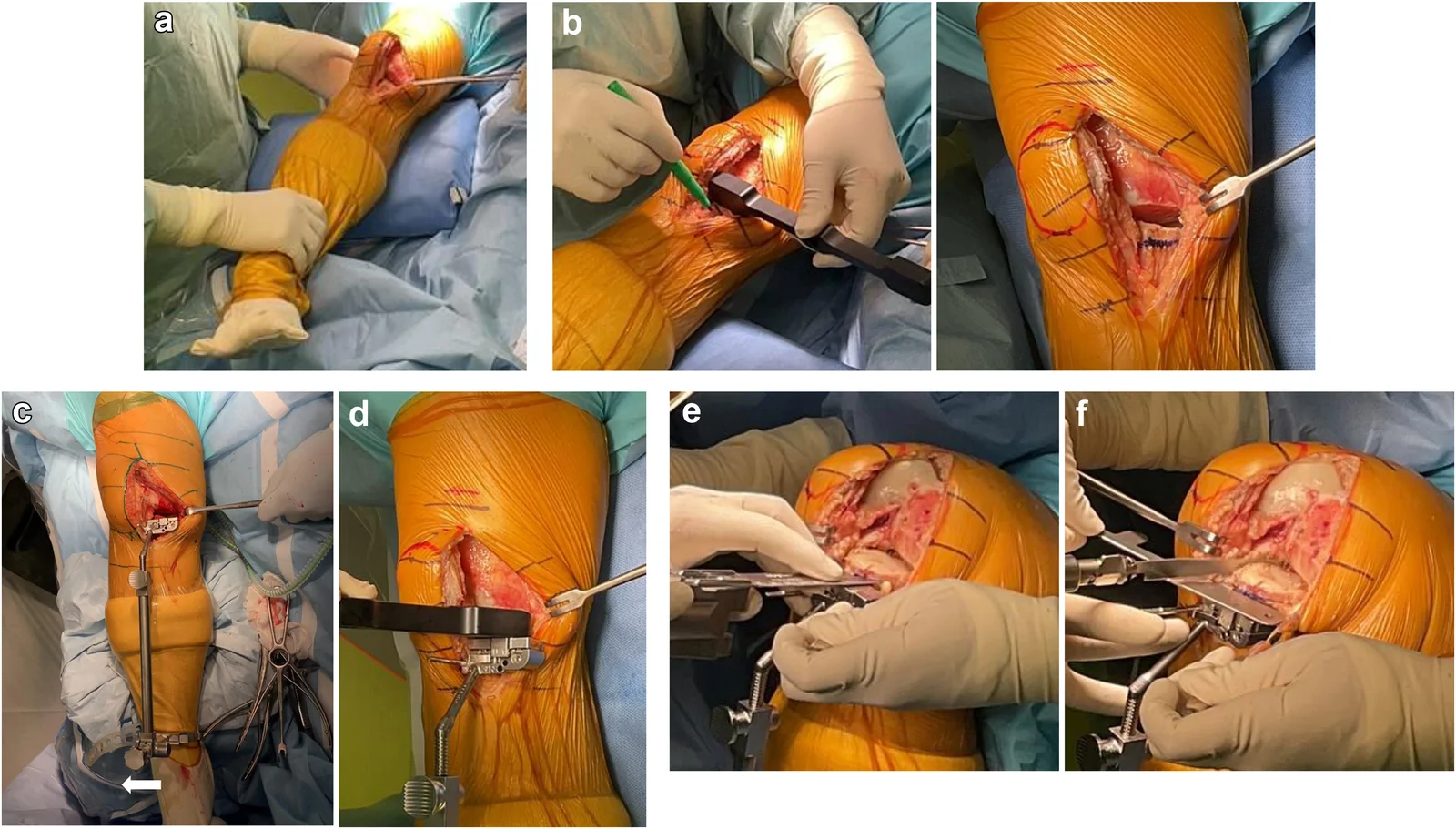
(a) Manual in-line traction is applied to the lower limb. (b) A reference mark is made 15 mm distal to the femoral distal cut surface to determine approximate tibial resection height. (c) Manual in-line traction is reapplied, and the distal portion of the tibial jig is slid until the tibial cut surface becomes parallel to the distal femoral cut surface. (d) Resection height is reconfirmed using a 15-mm spacer block. Tibial resection is performed carefully: (f) transverse cut and (e) vertical cut.

A reference mark is made distal to the femoral distal cut surface to estimate the initial tibial resection level. In the present implant system, this distance is 15 mm, corresponding to the combined thickness of the femoral component and polyethylene insert. This value should be adjusted according to the implant system used.

The proximal tibial cutting guide is positioned while maintaining the planned posterior tibial slope. Manual in-line traction is reapplied, and the distal portion of the tibial guide is adjusted until the planned tibial cut surface becomes parallel to the reconstructed distal femoral surface (Fig. 4c).

The resection level is confirmed using a spacer corresponding to the implant thickness before tibial resection. Transverse and vertical tibial resections are then performed (Fig. 4d-f).

### Spacer assessment and final preparation

The resected tibial bone fragment is measured using a caliper (Fig. 5a). The measured thickness is compared with the planned tibial component thickness and serves as a reference for determining whether additional tibial resection or adjustment of polyethylene insert thickness is required to reproduce the intended resurfacing thickness. Trial components and spacer blocks are inserted to evaluate the reconstructed joint space.

**Figure 5:**
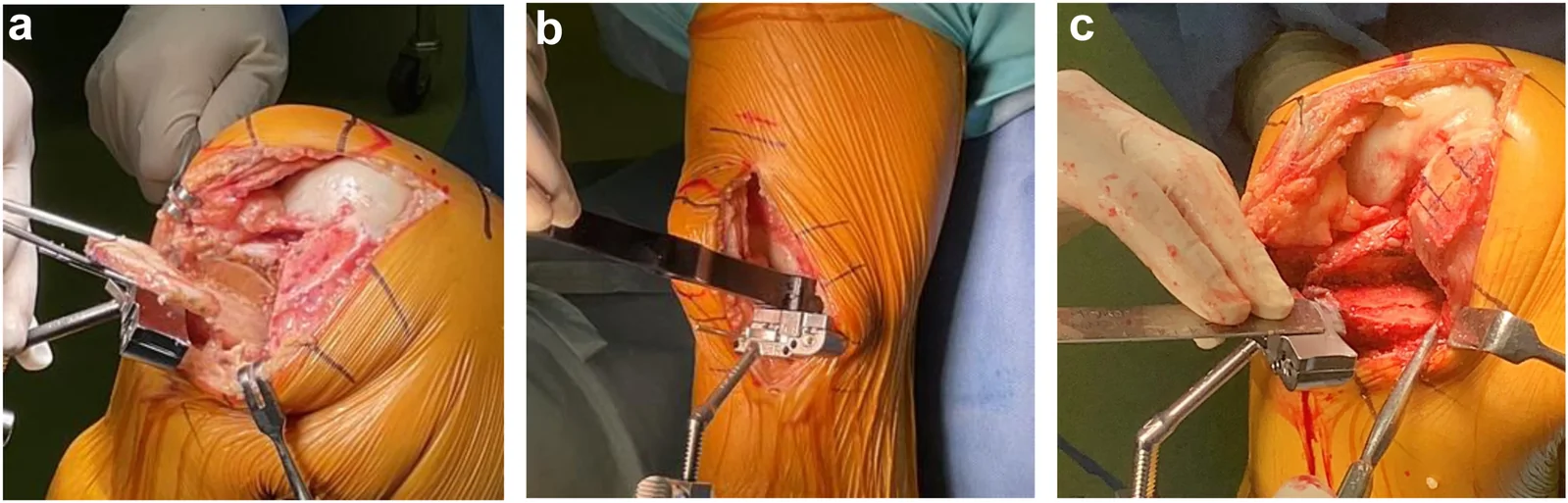
(a) The resected bone fragment is measured using a caliper. (b) The postresection space is checked with a spacer block. (c) Shim thickness and additional bone resection are adjusted until a 16-mm spacer block (to ensure a margin of 1 mm compared to the thickness of the implant of 15 mm) can be easily inserted and removed. If posterior slope is insufficient, additional adjustments are made as necessary.

Unlike conventional gap-balancing techniques, coronal balance is not assessed by creating a valgus stress gap. Instead, the relationship between the reconstructed femoral surface and tibial preparation is evaluated under manual in-line traction, which reproduces the physiological loading condition used during the procedure.

Trial assessment is performed with the knee in extension and throughout the range of motion to confirm smooth articulation, appropriate stability, and restoration of physiological ligament tension.

In the present implant system, a 16-mm spacer block provides a 1-mm clearance relative to the combined implant thickness of 15 mm. The appropriate spacer thickness is selected according to the implant system being used. If the trial construct is excessively tight or loose, adjustments are made by modifying tibial resection height, posterior tibial slope, or polyethylene insert thickness as appropriate.

The procedure is completed according to the manufacturer's recommended surgical technique.

Representative preoperative and postoperative radiographs are shown in Figure 6.

**Figure 6:**
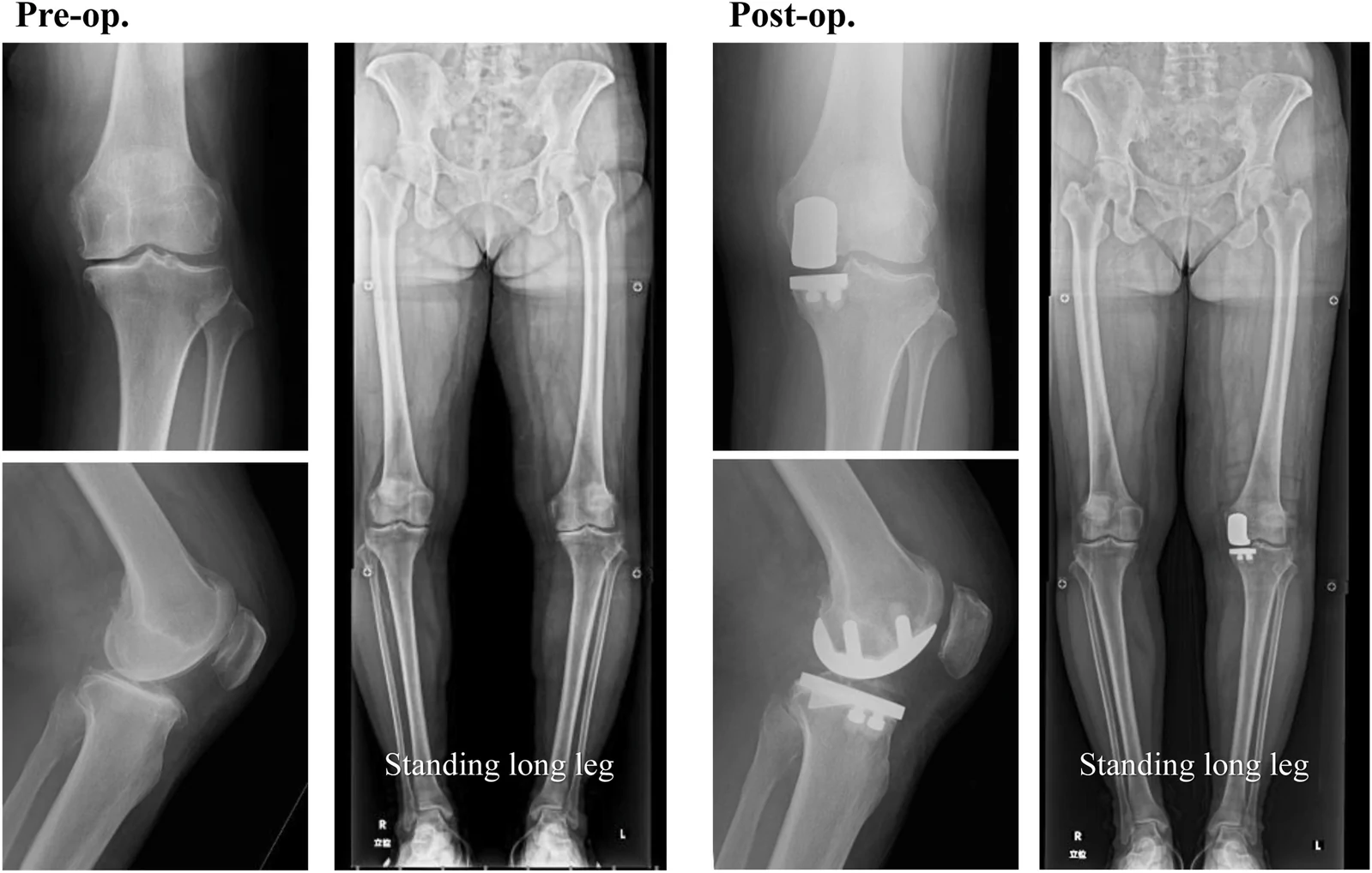
Preoperative and postoperative radiographs of a representative case.

## Discussion

The femur-first calipered kinematically aligned medial UKA technique described in this Technical Note applies the fundamental principles of calipered KA to fixed-bearing UKA. The primary concept of this technique is reconstruction of the femoral articular surface before tibial preparation, thereby establishing a stable anatomical reference for subsequent tibial resection.

Conventional UKA techniques have demonstrated excellent clinical outcomes and remain widely accepted. Although several studies have reported femur-first cut UKA [[15], [16], [17]] many commonly used workflows begin with tibial preparation, and femoral component positioning is subsequently influenced by tibial resection, gap assessment, and implant thickness. Although this approach has been successful clinically, the femoral component orientation may vary depending on the interaction between tibial preparation and intraoperative balancing techniques.

In contrast, the femur-first approach described in this study reconstructs the distal and posterior femoral anatomy before tibial preparation. This sequence is based on the concept that the femoral articular surface represents an important anatomical reference for restoring native tibiofemoral kinematics. By establishing femoral component position independently from tibial resection, the technique aims to reduce unnecessary variability in femoral orientation while maintaining patient-specific anatomy.

Recent studies have suggested that KA principles may be applicable to UKA and that femoral anatomical landmarks, including the medial wall of the medial femoral condyle, can provide reliable references for femoral component positioning. The present technique extends these concepts to fixed-bearing UKA by incorporating caliper verification of bone resection and femoral reconstruction before tibial preparation.

Another important feature of this technique is the method of intraoperative balance assessment. Unlike conventional gap-balancing approaches that frequently rely on varus-valgus stress testing to create symmetric gaps, this procedure uses manual in-line traction [[13], [14]] as the reference condition during tibial preparation and trial evaluation. This approach is intended to preserve the native relationship between the reconstructed femoral surface and the remaining soft tissues rather than to modify ligament tension to achieve predetermined gap values.

The technique also provides a reproducible method for determining tibial preparation parameters after femoral reconstruction. The posterior tibial slope is reproduced according to the native tibial surface morphology, and tibial resection height is adjusted based on the relationship between the reconstructed femur, trial components, and soft-tissue tension. Therefore, tibial preparation becomes an independent resurfacing step rather than the primary determinant of femoral component position.

Several limitations should be acknowledged. First, this is a technical description rather than a comparative clinical study, and the clinical advantages of this approach require further investigation. Second, although the principles are intended to be applicable to fixed-bearing UKA systems, specific instrumentation and implant dimensions may require adaptation according to the implant design. Third, appropriate patient selection remains essential, and this technique does not alter established indications or contraindications for UKA, including advanced lateral compartment degeneration, inflammatory arthritis, or instability requiring ligament reconstruction.

The purpose of this technique is not to replace established UKA methods but to provide an alternative anatomy-based workflow for surgeons interested in applying calipered KA principles to fixed-bearing UKA. By establishing the femoral reference before tibial preparation and evaluating the reconstruction under physiological loading conditions, femur-first calipered KA-UKA offers a reproducible resurfacing strategy that emphasizes restoration of patient-specific knee anatomy.

## Summary

Femur-first calipered kinematically aligned medial UKA is a surgical technique that applies the principles of anatomical resurfacing and patient-specific alignment to fixed-bearing UKA. By reconstructing the femoral articular surface before tibial preparation, this technique establishes a consistent anatomical reference for subsequent tibial resection.

The procedure uses caliper verification, trial component assessment, and manual in-line traction to evaluate the relationship between the reconstructed femur, tibial preparation, and preserved soft tissues. This workflow provides a reproducible approach to fixed-bearing UKA while maintaining the concept of restoring native knee anatomy.

Further clinical studies are required to determine whether this technique provides advantages over established UKA techniques. However, femur-first calipered KA-UKA represents an alternative resurfacing strategy that may improve consistency in femoral component positioning and intraoperative decision-making.

## Declaration of generative AI and AI-assisted technologies in the manuscript-preparation process

During the preparation of this work, the author (Yoshinori Soda) used ChatGPT in order to translate Japanese to English. After using this tool/service, the author reviewed and edited the content as needed and take full responsibility for the content of the published article.

## CRediT authorship contribution statement

**Yoshinori Soda:** Writing – review & editing, Writing – original draft, Supervision, Project administration. **Mitsuhiro Nakamura:** Validation, Methodology. **Toshiya Kano:** Validation, Methodology.

## Conflicts of interest

The authors declare there are no conflicts of interest.

For full disclosure statements, refer to https://doi.org/10.1016/j.artd.2026.102147.

## Appendix A Supplementary data

Conflict of Interest Statement for Kano

Conflict of Interest Statement for Nakamura

Conflict of Interest Statement for Soda
